# The use of animal models in preclinical investigations for the development of a surgical mesh for pelvic organ prolapse

**DOI:** 10.1007/s00192-024-05741-3

**Published:** 2024-02-15

**Authors:** Amelia Seifalian, Alex Digesu, Vikram Khullar

**Affiliations:** Department of Urogynaecology, Imperial College London, London, UK

**Keywords:** Pelvic mesh, Polypropylene, Animal model, Graphene, Preclinical trials, Biomaterials

## Abstract

**Introduction and hypothesis:**

Polypropylene (PP) mesh for the treatment of pelvic organ prolapse (POP) has raised substantial concerns over long-term complications, leading to its ban in multiple countries. In response, emerging materials are being explored as alternatives for prolapse surgery. Preclinical animal models have historically played a pivotal role in validating medical devices, prior to clinical trials. Successful translation of these materials necessitates the identification of suitable animal models that replicate the female human pelvis and its biomechanical properties. Preclinical in vivo testing assesses the safety of surgical mesh and treatment efficacy in preventing POP recurrence.

**Methods:**

The research critically reviews animal models used for preclinical pelvic mesh testing over the last decade and proposes a promising model for future preclinical studies.

**Results:**

Rats were the most common mammal used for toxicity and biocompatibility investigations through abdominal implantation. Although non-human primates serve as a gold standard for efficacy testing, ethical considerations limit their use owing to their close biological and cognitive resemblance to humans. Consequently, sheep were the most preferred large animal model owing to their reproductive system similarities and propensity for spontaneous POP following parity.

**Conclusion:**

The study contributes valuable insights into the selection of appropriate animal models for preclinical pelvic mesh testing, offering guidance that is crucial for enhancing the safety and efficacy of novel surgical interventions in the treatment of POP.

## Introduction

The use of animal models in clinical research traces back to ancient Greece and dates back to the sixth century BCE. Initially employed to enhance our understanding of clinical anatomy and serve as comparative models for studying mammalian anatomy, animal models have played a pivotal role in the evolution of scientific research. Over the centuries, the role of animal models has extended beyond anatomical studies to encompass a wide range of applications, including preclinical trials for testing medical drugs, surgical devices and even products in industries such as cosmetics. This has led to the animal studies industry becoming a billion-dollar market, with health care companies now relying on animal models to evaluate product safety, toxicity and efficacy prior to human translation.

**Fig. 1:**
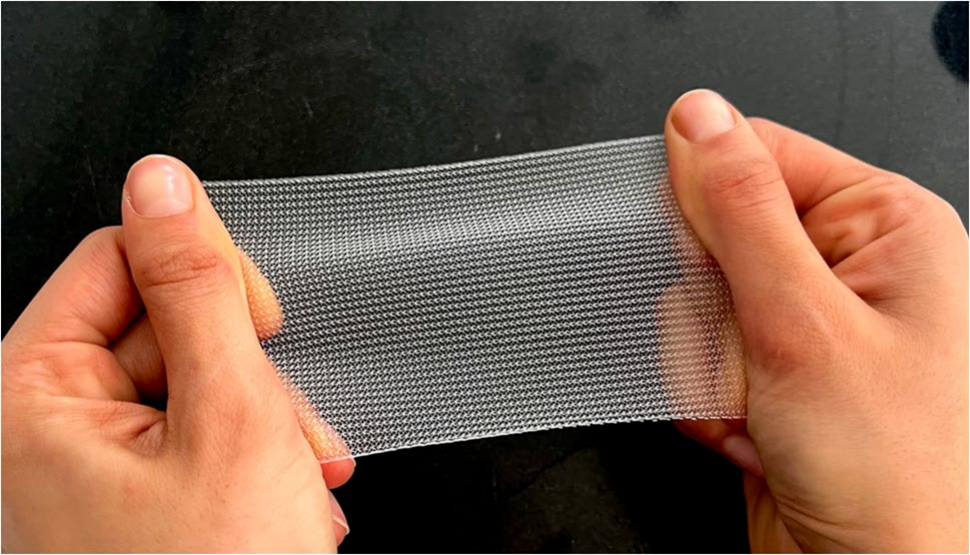
Polypropylene mesh used in pelvic organ prolapse surgery

The polypropylene (PP) mesh (Fig. 1) is a surgical adjunct used to treat pelvic organ prolapse (POP). The transvaginal PP mesh was initially approved in 1996, having been modelled from the mesh previously used in hernia surgery. However, concerns surrounding long-term complications, such as chronic pain and mesh exposure, have prompted the ban of transvaginal PP meshes in several countries, including the UK, USA and Canada. To introduce a novel surgical implant onto the market, extensive testing is imperative to ensure product safety prior to human use, involving meticulously designed preclinical trials. In this context, it becomes paramount to design appropriate animal trials and select the most suitable animal model that closely mimics the biomechanical properties and histology of the female human pelvis.

The primary objective of this research was to critically review the animal models used in preclinical pelvic mesh testing to date, aiming to propose the most suitable animal model for the design of future preclinical trials investigating novel surgical implants for prolapse surgery. Our review will highlight preclinical animal trials investigating the application of PP mesh in POP treatment over the last decade and assess the suitability of each animal model. In these in vivo trials, we aim to assess not only the safety of the mesh surgical adjunct in avoiding long-term complications but also its efficacy in preventing recurrence of POP.

## Materials and Methods

For this comprehensive review, we conducted a systematic search of three major databases: PubMed/Medline, Embase and Cochrane Library (Wiley). Our search was aimed at identifying relevant studies on animal models in POP research and the use of synthetic mesh. We employed the following key words: animal model; pelvic organ prolapse; pelvic mesh; synthetic mesh; and a range of animal species often used in preclinical trials. We restricted the search to publication over the last 10 years.

## Results

A summary of all preclinical trials utilising animal models to investigate the safety and efficacy of synthetic pelvic mesh has been highlighted in Table 1. Overall, we identified 36 animal trials dedicated to investigating synthetic mesh for POP in the past decade. In subsequent sections, we comprehensively assess the suitability of each animal model used.

**Table 1:** Demonstrating a summary of all the animal studies published to PubMed assessing the safety and efficacy of pelvic mesh implants in the treatment of pelvic organ prolapse in the last 10 years

Animal	Mesh	Site of insertion	Procedure	Follow-up (days)	Outcome	Comment	Country	Reference
Rat (Wistar)	Restorelle®	Anterior and posterior vaginal wall	PP mesh inserted via sacrocolpopexy in diabetes-induced and non-diabetic rats to assess inflammation. Following bilateral ovariectomy and supracervical hysterectomy	3, 7 and 42	Diabetes was associated with long-term inflammation, secondary to the dysregulated macrophage response	Diabetes is a known factor of inflammation, confirmed by results	USA	Liang et al. [1]
Pig (Yorkshire Crossed)	PP mesh	Vagina	Investigating the use of purified injectable exosome product to encourage tissue regeneration and treat ME. Meshes affixed with 2–0 PP sutures	35	Pigs treated with exosome injection and surgical correction saw highest incidence of ME resolution, followed by treatment with only exosome injection and no surgery	Note use of large pigs—70–80 kg. This was so that multiple meshes could be used per animal	USA	Kisby et al. [2]
Rabbit	PP mesh woven with thread of barium sulphate	Intraperitoneal flat inlay onto tissues	After 7 days, bloods were taken to assess acute toxicity. After 180 days, rabbits were euthanised to study histocompatibility and chronic toxicity	7 and 180	Adding radiopaque barium sulphate enables mesh visibility on X-ray imaging. Novel design is non-toxic and does not affect histocompatibility	Rabbits were suitable for testing toxicity and histocompatibility—no investigation for mesh efficacy	China	Li et al. [3]
Rat	PP mesh with pore sizes:	Abdominal wall	Plain textiles investigated using textile standards (NF-EN-13494–1). Mechanical characterisation performed using cyclic uniaxial tensile tests	90	Best outcomes with lighter mesh and low stiffness. Investigation into the effect of mechanical properties on native tissue	Results may not be suitable as the rat abdominal wall does not translate to the human pelvis	France	Morch et al. [4]
	A—1.3 mm; B—2.2 mm; C—3.9 mm							
Rabbit (NZW)	Implant A: human cadaver skin tissue, 10 × 5 mm	Subcutaneous abdominal wall and post submucosa layer of vagina	Surgical procedure, clinical complications intra- and post-operatively including pain variable and analysis of explants studied	180	PP mesh had 33% ME rate. At 180 days, 40% of the biologic implants showed degradation. Few complications for abdominal implants	Difficulty obtaining adequate blood sample and vaginal field too small	Spain	Peró et al. [5]
	Implant B: Gynaeband® PP mesh 10 × 5 mm and 5 × 5 mm							
Rabbit (NZW)	Mesh A: soft elastomer PDMS	Internal vagina	The mesh was implanted into the vagina following abdominal hysterectomy with preservation of the ovaries. Mesh A was heavier and less porous than PP mesh but with similar stiffness	84	Less negative reaction to mesh A with fewer complications and better structural morphology. Restorelle® was chosen as having least recorded functional and structural complications	Low stiffness important to prevent complications	USA	Knight et al. [6]
	Mesh B: Restorelle®							
Pig (Yorkshire Crossed)	Restorelle®	Midventral and midrostral region of denuded vagina	ME model used following treatment using exosome injection. Restorelle® chosen for being PP mesh with greatest porosity and least stiffness	84	The studies confirmed the efficacy of using an injectable exosome regenerative platform to treat ME. Multi-dose treatment resulted in better tissue regeneration	This team has performed two similar studies of this kind; this one tests dosages required	USA	Kisby et al. [7]
Rat (SD)	Mesh A: novel biomaterial, PCU, 3D printed crosshatch design 1-mm pores	Vagina	Supracervical hysterectomy and ovariectomy first performed, then mesh implanted	90	PCU has less stiffness and greater tensile strength than PP and is therefore less likely to result in mesh erosion and pain complications. Similar inflammatory response to both PCU and PP mesh	Small mesh constructs used as rats too small. Not placed under tension, which may affect results	USA	Bickhaus et al. [8]
	Mesh B: lightweight, knitted PP mesh 1.5-mm pores							
Rat (SD)	Lightweight PP meshes with 1.5-mm pore size	Vagina and proximal vs distal lumbar vertebrae	Comparison between sham operation only (control), mesh sutured only on the vagina (vaginal mesh), sacrocolpopexy without tension, and sacrocolpopexy with tension	90	Attachment of prolapse mesh resulted in an increased histological inflammatory response independent of tension	Ovariectomy to cancel hormonal effects on outcomes	USA	Bickhaus et al. [9]
Rat (Wistar)	Mesh A: biodegradable polymer 65% polycaprolactone and 35% polytrimethylene carbonate	Inter-fascial space between dorsal muscles (back)	Each rat had two meshes placed at the back, experimental and PP mesh, each 1 × 1 cm	90 and 180	Mesh A degraded by 9% over 6 months, noted to be too fast to allow tissue regeneration. More fibrosis noted with biopolymer matrix, which is ideal for managing POP	Biodegradable polymer not suitable for long-term prolapse support	Russia	Eisenakh et al. [10]
	Mesh B: PP mesh							
Rat (SD)	Gynemesh®	Vagina	Ovariectomy performed 1 week prior to mesh implantation. PP mesh seeded with human umbilical cord-derived stem cells	7, 28 and 84	At 12 weeks, better outcomes with stem cells seeded PP mesh with host response and tissue regeneration	Testing stem cells coating on larger animals would provide more accurate results	China	Deng et al. [11]
Rat (SD)	Mesh A: novel polycarbonate material based on fourfold hydrogen bonding ureidopyrimidinone	Abdominal wall	Abdominal wall was reconstructed with mesh in the rat hernia model	2, 7, 14, 28 and 90	Results showed that mesh A resulted in better tissue integration and ingrowth, with reduced scar formation	The results are unlikely to be accurate as the rat abdominal wall hernia model was used, which does not translate to vaginal prolapse	European	Mori da Cunha et al. [12]
	Mesh B: pristine-based mesh							
Sheep	Mesh A: polyamide-based mesh; B: same as A but dip-coated in gelatin and stabilised with 0.5% glutaraldehyde; C: same as B, but seeded with autologous ovine eMSCs	Posterior vaginal wall, rectovaginal space	Subtotal hysterectomy via ventral midline laparotomy 4 weeks prior to mesh implantation. Mesh implantation via transvaginal surgery of the posterior vaginal wall	30	Mesh A had the best outcomes, then mesh C. eMSCs had a role in replenishing and retaining muscle cells surrounding the uterus. eMSCs in mesh C resulted in better histological outcomes and tissue integration than in mesh B	This study tested implants for biocompatibility and toxicity, as well as treatment efficacy by measuring improvement in POP using POP-Q measurements	Australia	Emmerson et al. [13]
Rat (Wistar)	Type 1 PP monofilament macroporous mesh	Abdominal fascia and muscles in the four corners of the abdominal wall	Vertical abdominal cutaneous incision and abdominal fascia dissection. Measuring ideal endpoint for animal study follow-up	30, 60, 90, 120 and 150	Mechanical properties of implants evolve over time as they integrate with native tissue. In this situation—abdominal fascia of rats, mechanical properties stabilised after 2 months	Extremely useful research for the design of animal studies, 2 months proposed as a suitable minimal endpoint	France	Doucède et al. [14]
Sheep	Mesh A: titanised PP lightweight mesh—TiLOOP	Rectovaginal septum	Posterior vaginal wall dissected from the perineal body to the vaginal apex along the midline. Mesh implanted between the rectum and the vaginal epithelium	7 and 84	Mesh A showed less inflammation at 1 week. At 12 weeks, there was no significant difference between the biomechanical properties of mesh A and mesh B	The research team was able to follow this study with a clinical trial	China	Ai et al. [15]
	Mesh B: Gynemesh®							
Rabbit (NZW)	Restorelle®	Anterior and posterior vagina	Mesh implanted into the anterior and posterior vagina via lumbar colpopexy after hysterectomy, with preservation of the ovaries	84	Rabbit is similar enough to cautiously use for histological studies for POP, noting differences	Increased availability of rabbits and cheaper, good alternative	USA	Knight et al. [16]
Mouse	Mesh A: biodegradable PCL, aloe vera-sodium alginate hydrogel and eMSCs	Abdominal wall	Following sharp dissection of the abdominal wall, blunt dissection was used to create a subcutaneous pocket to insert the mesh	7	Good biocompatibility and tissue integration. The eMSCs were retained until endpoint. Mesh A, which included eMSCs, had most positive outcomes	Short-term test on smaller animal unlikely to translate to human studies	Australia	Paul et al. [17]
	Mesh B: PCL construct							
	Mesh C: Mesh A without eMSCs							
NHP	Mesh A: Gynemesh® + two-ply MatriStem	Anterior/posterior vagina wall and longitudinal ligament of the sacrum	MatriStem is an extracellular matrix bioscaffold derived from urinary bladder matrix. Mesh is implanted via sacrocolpopexy following a hysterectomy	90	Mesh B had poor outcomes of vaginal atrophy and reduced vaginal smooth muscle contractility; mesh A attenuated this impact. Mesh C had increased vaginal smooth muscle but no other significant changes	Use of gold-standard non-human primate supports the accuracy of the results	USA	Shaffer et al. [18]
	Mesh B: Gynemesh®							
	Mesh C: six-ply MatriStem scaffold							
Sheep	Mesh A: novel mesh of bacterial cellulose	Midline between rectum and vaginal epithelium	Mesh was smoothed prior to implantation to prevent any folds Bacterial cellulose mesh is developed via fermentation of *Acetobacter xylinum* with natural ingredients including coconut water and sugar cane molasses	7 and 84	Mesh A induced a greater inflammatory response than the comparator at both endpoints. Biomechanical properties met minimal requirements to treatment of POP, but no improvement compared with mesh B	The outcomes were not promising for the use of mesh A to treat POP; other alternatives currently at the same stage have improved outcomes	China	Ai et al. [19]
	Mesh B: Gynemesh®							
Rat (Wistar)	Mesh A and B: PCL—2 doses of fibroblast growth factor	Abdominal wall	The PCL meshes were fabricated via electrospinning then coated with a hydrogel containing either fibroblast growth factor or connective tissue growth factor. Mesh A and B: hollow fibre. Mesh C, D, E and F: solid fibre	56 and 168	High-dose fibroblast growth factor did not improve collage formation. Hollow fibre mesh underwent total degradation at 24 weeks compared with solid fibre. Mesh E including stem cells was the only mesh not causing complications and had best biomechanical outcomes	Number of mesh studies in rat models but ideally a larger animal would be used to test treatment with more than one mesh per animal	Denmark	Hansen et al. [20]
	Mesh C and D: PCL—with and without fibroblast growth factor							
	Mesh E and F: PCL and connective tissue growth factor—with and without rat mesenchymal stem cells							
Mouse	Meshes A: poly(L-actic acid)-co-PCL; B: mesh A + gelatin; C: mesh A + eMSCs; D: mesh A + gelatin + eMSCs	Abdominal wall	Mesh implanted via longitudinal skin incision of lower abdomen. Two mesh inserted per animal	7 and 42	All mesh biomechanical properties were satisfactory. Mesh D with gelatin and eMSCs had the best outcomes in terms of anti-inflammatory response and tissue integration. Combination with gelatin increased retention of eMSCs	Biomechanical properties would not be translatable as mice are much smaller than humans and have different mechanical properties	Australia	Mukherjee et al. [21]
Rat and rabbit	Mesh A: PCL modified with ureidopyrimidinone	Rat: left hemi-abdominal wall Rabbit: right lower and left upper quadrant	Rat: herniation made in abdominal wall Rabbit: each rabbit is implanted with two meshes in abdominal wall	Rat: 7, 42 and 54 Rabbit: 30 and 90	In both animals, compliance of mesh A was similar to that of native tissue. In rats, mesh B was stiffer. Foreign body giant cells were present increasingly in tissues where degradation had taken place	Would be better to separate papers for each animal to avoid confusion regarding results	European	Hympanova et al. [22]
	Mesh B: Restorelle®							
Sheep	Mesh A: Restorelle®	Rectovaginal septum, 3 cm from the hymenal ring	Posterior vaginal wall surgery took place with mesh inserted at the rectovaginal septum	60 and 180	Mesh B had partially degraded in 90% of sheep at 180 days but meshes A and C remained fully intact. Biomechanical properties were similar amongst all groups	Biodegradable mesh had degraded by the endpoint, as would be expected; however, biomechanical properties similar amongst groups	European	Hympánová et al. [23]
	Mesh B: biodegradable ureidopyrimidinone-polycarbonate							
	Mesh C: non-degradable polyurethane							
Rat	Mesh A: standard weight PP mesh 72 g/m^2^	Abdominal wall	Each rat had two meshes inserted on either side of the midline incision of the abdominal wall	4 and 30	There was no significant difference in outcomes for mesh A and mesh B	Similar results may be due to a lack of longer-term follow-up	Brazil	Bronzatto and Riccetto [24]
	Mesh B: lightweight PP mesh 16 g/m^2^							
Rat	Mesh A: polylactic acid and PCL microfibres electrospun onto PP mesh	Onlay position in abdomen	Meshes were processed as described prior to implantation into the rat model, in abdominal subcutaneous tissue	14 and 28	Mesh A had better outcomes in terms of tissue regeneration and adhesion resulting in integration. The thickness of mesh A increased most at both endpoints, likely because of tissue growth on the surface	Investigation of the fabrication method of two meshes. Analysis lacks robustness, with conclusions made on assumptions	China	Lu et al. [25]
	Mesh B: PP mesh immersed in polylactic acid and PCL solution							
Rat (SD)	Mesh A: Gynemesh®	Posterior vaginal wall	Initially, subjects underwent ovariectomy 1 week prior to mesh implantation. Note that meshes were prepared with either human umbilical mesenchymal stem cells or smooth muscle cell-differentiated stem cells	7, 28, 56 and 84	Meshes B, C and D had better outcomes than mesh A. There were thicker layers of tissue growth on all meshes with stem cells. Mesh D had the most promising results	Consideration of the accuracy of results with implantation of human stem cells into the rat model	China	Ding et al. [26]
	Mesh B: mesh A and human umbilical cord stem cells							
	Mesh C: mesh A and smooth muscle cell-differentiated stem cells							
	Mesh D: mesh C and human umbilical cord stem cells							
Sheep	Mesh A: PVDF mesh with arms	Beyond rectovaginal septum	MR imaging was taken of mesh in vivo to investigate mesh changes in shape and geometry following implantation	2, 14 and 60	Mesh A had the least surface area decrease compared with mesh B, occurring immediately post-operatively. Mesh size was mostly stable afterwards	Novel methods assess the outcomes of significant mesh shrinkage	Belgium	Iva et al. [27]
	Mesh B: flat PVDF mesh							
Rat (SD)	Mesh A: PCL modified with ureidopyrimidinone	Abdominal wall defect	Full-thickness abdominal defect created then repaired and reinforced with mesh	7 and 42	Mesh A does not compromise physiological compliance and was not fully degraded at 42 days. Mesh B’s biomechanical properties remain far from physiological compliance	Biomechanical properties were recorded; however, the rat model was not translatable to the human vagina for prolapse	European	Hympanova et al. [28]
	Mesh B: Restorelle®							
Rabbit (NZW)	Mesh A: knitted design pure polylactic acid	Four quadrants of abdominal wall—onlay position	Each subject had four meshes implanted, two of each type on either side of the midline incision	30, 90 and 180	Biocompatibility was similar in both meshes. Surrounding tissue to mesh A recovered better than that to mesh B. There was increased tissue regeneration and reduced shrinkage with mesh A	It was recognised that repeating the experiment in a sheep model would provide more accurate results	Australia and China	Lu et al. [29]
	Mesh B: Surgimesh® Prolapse, lightweight and large pore PP mesh							
NHP	Six-ply MatriStem scaffold	Vagina	All animals underwent hysterectomy and complete transection of uterosacral ligaments and paravaginal attachments to the pelvic sidewall prior to the procedure. Mesh implanted either via the transvaginal or the transabdominal route	90	Good overall biocompatibility was recorded. Transvaginal insertion resulted in poorer outcomes, adjacent to the site of incision	No active control group. Results from a previous study were used for sham comparison. Note the gold standard animal model	USA	Liang et al. [30]
Rat (Wistar)	Mesh A: Parietex Composite® Covidien multifilament polyester mesh	Between the posterior cervix and the anterior longitudinal ligament of the lumbar vertebrae	Mesh A was fabricated using polyethylene terephthalate coated with porcine collagen-polyethylene glycol and glycerol	14	Mesh A had better adhesion outcomes; however, it also caused a more pronounced host inflammatory response and foreign body reaction	Note that the strong host inflammatory response in mesh A was anticipated	Turkey	Gokmen-Karasu et al. [31]
	Mesh B: Surgipro® macroporous multifilament PP mesh							
Rat (Wistar)	PCL and polyethylene oxide	Abdominal wall	Mesh combined with basic fibroblast growth factor versus mesh implantation. Mesh implanted at the site of full-thickness fascia-muscle defect	28, 56 and 168	Growth factors prevented degradation of mesh for 28 days. Positive outcomes recorded but degradation occurred too fast to support tissue regeneration	Longer follow-up would have allowed assessment of the full degradation of the implant	Denmark	Glindtvad et al. [32]
Rats (Wistar)	Mesh A: heavy weight PP mesh 62 g/m^2^	Abdominal wall	Mesh implanted between the hypodermis and abdominal muscular fascia. Mesh A as per the standard procedure. Mesh B was implanted in both the transverse plane and the longitudinal plane	7, 30 and 60	Implanting mesh B in the transverse plane exhibited similar outcomes to mesh A. Less stiffness and maximum load were seen with longitudinal plane implantation	This research confirms that physical and textile implant properties greatly effect clinical outcomes	Brazil	Bigozzi et al. [33]
	Mesh B: lightweight PP mesh 16 g/m^2^							
Rabbit (NZW)	Mesh A: 1 × 1 cm PP mesh	Abdominal wall	Mesh implanted between the hypodermis and abdominal muscular fascia. Collagen I, II and inflammatory infiltrate levels were analysed at implant site	7, 30 and 90	Coating PP mesh with platelet-rich plasma enhanced tissue regeneration with higher total collagen concentration	Promising results; however, it is unclear if these histology outcomes will result in better clinical outcomes	Brazil	Avila et al. [34]
	Mesh B: mesh A coated with platelet-rich plasma extracted from blood via centrifuge							
Rabbit	Mesh A: PP mesh	Abdominal wall	Meshes C and D were compared as alternative materials to commercially available Meshes A and B. Implants were placed in full-thickness abdominal wall defects	30 and 90	Meshes C and D had better outcomes, with decreased inflammatory response	Promising results regarding alternative materials; however, these would need replication in larger animal models	Belgium and UK	Sabiniano et al. [35]
	Mesh B: PVDF mesh							
	Mesh C: polyurethane mesh							
	Mesh D: poly-L-lactic acid							
Sheep	EndoFast Reliant™ System	Thigh fascia	EndoFast Reliant™ System was used to insert mesh into sheep thigh fascia. Pullout force was measured at each endpoint	0, 3, 7, 15, 30 and 45	Test to investigate the strength of attachment; results showed that the experimental system provided much stronger mesh attachment than trocar-based methods	Sheep thigh was used as the site of implantation owing to histological similarity, rather than vagina tissue	Israel	Alcalay et al. [36]

Only papers written in English language were included in the table

*eMSCs* endometrial mesenchymal stem cells, *ME* mesh exposure, *NHP* non-human primate, *NZW* New Zealand White, *PCL* poly ε-caprolactone, *PCU* polycarbonate urethane, *PDMS* polydimethylsiloxane, *POP* pelvic organ prolapse, *PP* polypropylene, *PVDF* polyvinylidene fluoride, *SD* Sprague–Dawley

### Mouse

Mice are small, readily available and cheap, and are hence commonly used in preclinical trials. However, their suitability depends on the purpose of the trial. Owing to their small size, housing is easy. Mice may be used initially to investigate the toxicity of the implant material prior to testing efficacy in larger animal models.

The mouse reproductive tract differs from the human reproductive tract in a number of ways. A significant structural difference is that midline fusion of Müllerian ducts leads to a unicornuate uterus in humans and a bicornuate uterus in mice [37]. Researchers must be mindful of these anatomical differences when designing experiments or interpreting results, especially if the study involves aspects of the reproductive tract that are influenced by uterine structure. This structural difference makes mice unsuitable for the mechanical investigation of a novel surgical adjunct for POP.

Of all 36 trials, only two of the trials of the last 10 years used mice for preclinical tests. Both of the preclinical trials that took place in mice investigated the use of endometrial stem cells to enhance tissue regeneration and recovery [17, 21]. The findings of both concluded that endometrial mesenchymal stem cells improved clinical outcomes and that gelatin improved retention of these stem cells. Mice were deemed most suitable for these studies as the breed of mouse used (NOD SCID gamma mice) lacks an adaptive immune system and allows focus on the innate foreign body response. In addition, this breed has a longer retention period of stem cells.

### Non-Human Primate

Non-human primates (NHPs) are the gold standard animal model for preclinical trials investigating a surgical adjunct for POP repair surgery. NHPs, especially rhesus macaques, share several anatomical and physiological similarities with humans, making them an appropriate model for studying pelvic anatomy. For example, their bipedal walking, squatting behaviour on defecation and vaginal delivery of live infants are characteristics that closely resemble those of human activities. Thus, the NHP is an appropriate model for investigating the efficacy of a pelvic implant.

The rhesus macaque is an NHP species considered most similar to the female human with regard to pelvic anatomy and mechanical properties. Rhesus macaques experience pelvic remodelling during pregnancy, primarily because of the larger head size of their infants—similar to humans [38]. This remodelling process, particularly in multiparous individuals, increases their susceptibility to spontaneous POP. This characteristic makes them even more relevant for POP research.

Two animal studies of the last 10 years used NHPs, experimenting with commercial PP mesh (Gynemesh™) and MatriStem as treatments for POP [18, 30]. Both the aforementioned studies used the rhesus macaque species for their investigations. Both these studies were carried out by the same research team and the research has not yet been brought forward into clinical trials. No results have yet been found investigating the use of MatriStem to treat POP.

Despite their suitability as animal models for preclinical trials, NHPs are less commonly used in research owing to several challenges. These include high cost, difficulty in obtaining and housing, and ethical concerns associated with use in biomedical research. In summary, although NHPs provide a valuable and relevant model for investigating the efficacy of pelvic implants for POP repair, their use is limited by practical and ethical considerations. Nonetheless, their anatomical and physiological similarities to humans make them a valuable resource for understanding and developing treatments for POP.

### Ovine

Ovine, or sheep, are large animals abundant and readily available from the farming industry, making them relatively affordable as a model for large animal studies. Their availability and cost-effectiveness contribute to their frequent use in research. Sheep are unique among quadrupedal mammals in that they can develop spontaneous POP. This phenomenon is believed to be related to their delivery of large live infants [39, 40]. Understanding and investigating spontaneous POP using sheep can be crucial to providing insights into treatment and cure in humans.

**Fig. 2:**
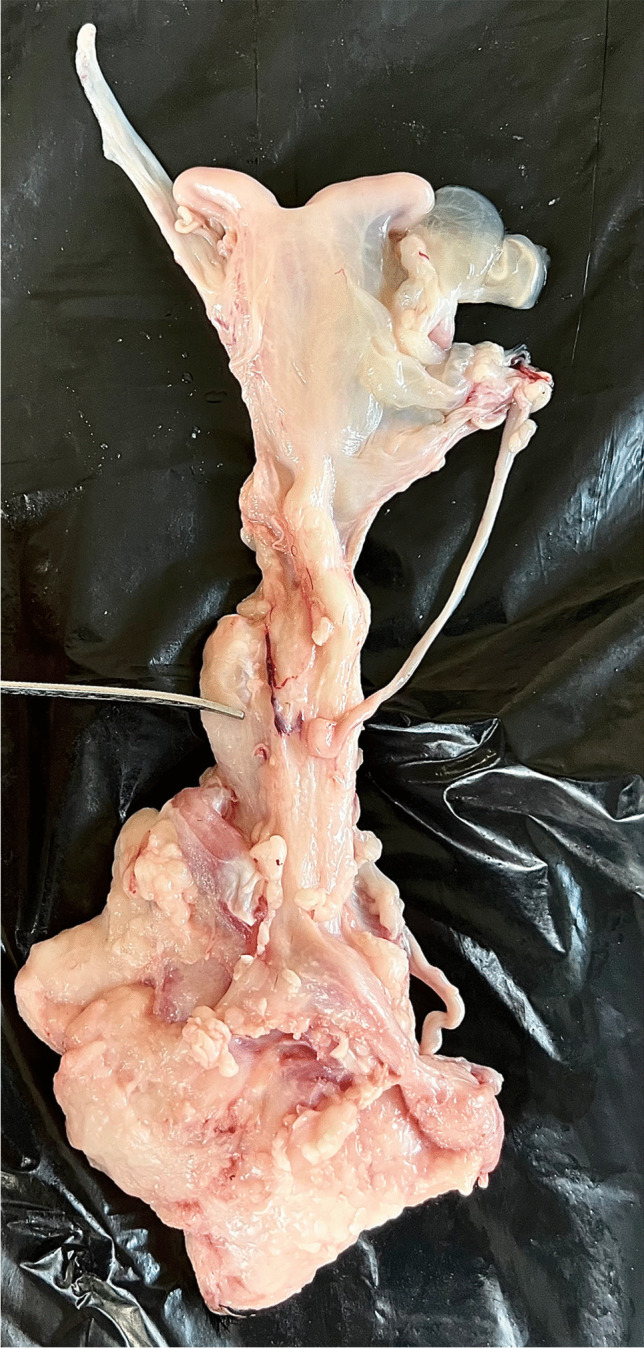
Reproductive system of a sheep obtained from an abattoir

Ovine pelvic anatomy is considered most similar to that of humans compared with other quadrupedal mammals [22]. This similarity extends to histology, vaginal size and collagen composition [41]. This makes sheep a valuable model for studying POP in relation to human anatomy. Figure 2 depicts the sheep reproductive system, showing the uterine horns and body, the cervix as pointed out by the instrument, leading inferiorly to the vagina.

Sheep were the most popular large animal model, with 7 of the 36 animal trials noted in Table 1 being performed in sheep. These trials likely chose sheep because of their anatomical similarities to humans and their propensity to develop POP. However, this does not mean that working with sheep as an animal model does not carry its own downsides and challenges.

One challenge associated with using sheep in preclinical trials is the difficulty in anaesthesia and positioning. Sheep have a non-straight back, which goes on to complicate surgical procedures when placing these animal models in a dorsal position; to overcome this the procedure would have to be performed as fast as possible whilst maintaining accuracy [42]. In addition, continuous gastric secretions during the procedure cause problems, with the possibility of post-operative complications. It is important to consult a veterinary specialist prior to performing any procedure.

One particularly promising aspect of using sheep as a model is that some research groups have successfully transitioned from preclinical trials in sheep to clinical trials in humans. A research group in China carried out initial investigations in the ovine model and continued to human clinical trials using their titanium-coated PP mesh, TiLOOP [43]. Note that this was the only animal study of a novel implant for POP identified to have been carried on into human clinical trials. This suggests that the sheep model has translational potential for preclinical studies.

In summary, ovine models offer anatomical and physiological similarities to humans, making them valuable in preclinical trials for POP treatments. Although there are challenges related to anaesthesia and positioning, the translational potential demonstrated in some studies underscores the significance of this animal model in advancing POP research.

### Porcine

Porcine or pig models have been among the least commonly used animal models for preclinical trials investigating surgical adjuncts for POP repair surgery. The porcine model was used for only 2 of the 36 studies identified in Table 1, which is the same frequency as the use of the mouse model and NHPs [2, 7]. These studies were both carried out by the same research group. One advantage highlighted by the research group was the larger size of the pig vagina. This larger size allowed them to utilise several implants per animal for their studies. This can be advantageous for particular experimental set-ups, especially when the quantity is significant.

A notable challenge associated with the use of pigs is their growth pattern. Pigs continue to have growth spurts until sexual maturity is reached, which can create additional costs and logistical challenges related to handling and housing. This continuous growth can affect the stability of mechanical properties, potentially impacting the reliability of the study results. Owing to the challenges related to growth and associated costs, pigs are generally considered less desirable for research purposes. The abundance of pigs in the farming industry does not make them the preferred choice for preclinical studies.

In summary, although porcine models have been used in a limited number of preclinical studies for investigating POP treatments. Their growth pattern and associated challenges make them less desirable for this purpose than other animal models, such as sheep. Researchers need to consider the potential impact of growth-related variability on study outcomes when using porcine models in such surgical research.

### Rabbit

The rabbit serves as a valuable animal model for conducting initial toxicology and biocompatibility studies related to surgical implants for POP. However, its essential to note that rabbits are not known to suffer from POP and therefore the rabbit model is not an appropriate model for testing treatment efficacy of a novel implant [44]. Despite this, rabbits have some desirable qualities in that they are relatively larger in size than other rodents, such as rats [5]. The larger overall size of rabbits provides researchers with a more feasible platform for investigating POP grafts.

The rabbit has an internal abdominal vagina and an external vagina. The external vagina is better accessible for surgical procedures requiring pelvic access. Rabbit was used in only 8 of the studies outlined in Table 1. Because rabbits are larger than rats, vaginal implantation was observed to be the main benefit [5]. However, it is noted that of the 8 studies performed in rabbits, only 3 of these studies opted to use the vaginal site to investigate the mesh graft [5, 6, 16]. Future research may explore methods of using rabbit models in ways that benefit researchers and allow reliability of results, by way of testing biocompatibility and toxicology at the site of vaginal tissue.

### Rats

Rats emerged as the most frequently used animal model, featured in 17 out of the 36 trials. This is due to a number of advantageous and desirable qualities of the rat model. Rats are small and do not grow significantly, are thus cost-effective and easy to feed, handle and house, and do not require much space. Rats are also readily available, thus overall providing a practical option.

The small size of the rat model does make it an economical choice, as smaller sections of implant material can be investigated per animal model. However, this prevents investigation of efficacy. The small size of rats also imposes limitations on the level of tension that can be applied when investigating pelvic mesh, thereby restricting the scope of investigations [10]. Although the rat model cannot be used to test treatment efficacy, it is readily available for toxicology and biocompatibility.

Rat connective tissue composition is similar to that of humans [44]. Other research has found the rat model more suitable for pelvic floor studies than mouse or rabbit, because there are more similarities to the human pelvis [45]. A common site on the rat model for device implantation is in the subcutaneous tissue of the abdominal wall [46]. This site provides an easier surgical procedure and a large surface area for explant analysis.

In conclusion, rats serve as valuable subjects in the early stages of preclinical trials for the investigation of new materials. Desirable qualities include affordability, accessibility, and tissue composition similarities to humans. Rats provide a suitable benchmark for initial studies of material toxicity and biocompatibility, prior to continuing preclinical tests of treatment efficacy in larger animals.

## Discussion

The human pelvis has unique features that support upright bipedal transport, which thus has a significant impact on pelvic environment. These features play a crucial role in designing preclinical trials to assess the efficacy of surgical implants for treating POP. Replicating the biomechanical environment of the human pelvis closely in in vivo investigations is essential for ensuring successful human translation. NHPs are considered the gold standard animal for these preclinical trials, but their use is limited owing to high costs and ethical concerns. Preclinical guidelines categorise NHPs as “acutely scarce resources” [30]. Therefore, academic and industry researchers must conduct investigations using alternative suitable models.

The “3Rs” principle, developed over 50 years ago, provides a framework for responsible animal research. The principle advocates the reduction, replacement and refinement of animals included in preclinical in vivo investigations. Reduction translates to using the minimum number of animals for consistent results. Refinement means not causing the animals any unnecessary harm. Replacement is to replace animals with other modes of investigation, such as numerical and computer modelling [47]. This framework is aimed at minimising animals used whilst ensuring consistent and reliable results. An alternative, in order to reduce and refine the use of animal models, would be to encourage further use of ex vivo studies prior to in vivo investigations.

The choice of the animal model for preclinical investigations should align with whether the focus is on biocompatibility/toxicity studies versus treatment efficacy. Rats are a common animal model for biocompatibility and toxicity studies, across all specialities. This is because they are small and easy to house and maintain. Only the material is being investigated; therefore, a small amount can be implanted subcutaneously. Owing to the frequent usage of rats in biocompatibility studies, established protocols exist providing consistency for biocompatibility and toxicity studies.

The anatomical structures and organ sizes are important to prepare for when planning animal trials. The adult human uterus measures approximately 8cm in height, 5cm in width and 3cm in thickness, with variations amongst individuals, parity, and stage of the menstrual cycle. Smaller animals, such as mice, have much smaller pelvic dimensions, therefore limiting the amount of material available for use in the model. Therefore, efficacy investigations require large animal models.

With regard to efficacy, sheep models provide the most suitable large animal model for efficacy investigations. A degree of error needs to be considered owing to bipedal versus quadrupedal locomotion. In addition, most animals, including sheep, are structured to support tails and tail function with muscles pointed dorsally, converse to the human pelvis [48]. The sheep have been recorded to suffer from spontaneous POP, as mentioned above, and so provide a useful, replicable environment for POP investigation.

## Conclusions

In this paper, we performed a thorough investigation of the animal models used to investigate a surgical adjunct for the treatment of POP. Six animal models were identified from preclinical trials spanning over the last decade, including mice, non-human primates, pigs, sheep, rabbits, and rats. Each animal model was discussed in detail highlighting the benefits and downsides of use. We concluded that rats were the most frequently used species, owing to their small size and the fact that they are readily available. The gold-standard animal model is the non-human primate; however, this is rarely used in reality owing to ethical concerns and limited availability. Sheep were the most common large animal model, as they provide a suitable alternative and are known to develop spontaneous POP. Preclinical trials are critical to evaluating the safety and efficacy of a device prior to human translation and careful selection of the animal model and design of the trial is significant to the translatability of outcomes.

## Data Availability

Data sharing is not applicable. No new data were created or analysed in this study. Data sharing is not applicable to this article.
